# Report on the Chief Results Obtained by the Use of the Microscope in the Study of Human Anatomy and Physiology

**Published:** 1843-04

**Authors:** 


ART. XI.—*Report on the chief Results obtained by the use of the Microscope in the study of Human Anatomy and Physiology.* By JAMES PAGET, Demonstrator of Anatomy at St. Bartholomew's Hospital. —*London,* 1842. 8vo, pp. 51.

As this small work is merely a reprint, with additions, of a paper which appeared in our Number for July last, we only notice it here for the purpose of stating that the additions are of the same sterling value as the original text, and therefore that the Report as it now stands, must be regarded as an improved edition of the original. We believe we only echo the opinion of the best judges in saying that Mr. Paget's Report is a master-piece; evincing, in a striking manner, the author's industry, learning, and good sense, and bearing on its face the unmistakeable impress of truthfulness of statement and soundness of inference.

